# Early-Onset Polyarticular Gout: A Case Report

**DOI:** 10.7759/cureus.90199

**Published:** 2025-08-15

**Authors:** Lara Kose

**Affiliations:** 1 Department of Medicine, University of California Los Angeles David Geffen School of Medicine, Los Angeles, USA

**Keywords:** acute gout attack, gout, gout chronic kidney disease, hyperuricemia, polyarticular arthritis

## Abstract

Gout is a form of arthritis involving the inflammatory response to the deposition of monosodium urate crystals. This is a case of a 39-year-old male who presents with significant polyarticular gout with a serum urate level of 10.2 mg/dL. He was considered to have early-onset polyarticular gout. Risk factors include comorbid hypertension and chronic kidney disease. Challenges arise when considering treatment options given these comorbidities while aiming to manage the patient’s symptoms effectively.

## Introduction

Gout is the most common inflammatory arthritis. It affects approximately 12.1 million adults in the United States, with a prevalence of 5.1%. It involves intra-articular deposits of monosodium urate crystals in the joints, bones, and soft tissue. Patients may experience painful, red, swollen, tender joints and/or tophi. The potential associated mechanism may be excessive production of uric acid and/or the inability to efficiently excrete it. Crystal formation occurs in the presence of persistent hyperuricemia when soluble urate levels consistently exceed the saturation threshold of 6.8 mg/dL, above which it crystallizes at 35°C [1,2]. When symptom onset occurs before the age of 40, it is considered early-onset [3]. When multiple joints are involved, gout may or may not be considered the most likely diagnosis, given the lower prevalence. This became evident with the 2015-2016 National Health and Nutrition Examination Survey (NHANES) cross-sectional survey in the United States. In the 20 to 39 year age group, there was a prevalence of 0.7% (95% CI 0.3; 1.5) [3].

## Case presentation

A 39-year-old male with a history of hypertension and prediabetes presented to the outpatient clinic with chronic multiple joint pain intermittently over the past two months. This involved the wrists, elbows, and knees. Throughout this time, he had experienced episodes of debilitating joint stiffness. Symptoms were exacerbated by physical activity and were self-limiting until recently. He reports a diet high in red meat intake. Upon arrival at the clinic, he had an exacerbation of right knee pain with edema for four days without any inciting event or injury. He was unable to bend the knee or bear weight. On exam, there was evidence of an effusion in the right knee and mild erythema overlying the first metatarsophalangeal joint. The serum urate level was 10.2 mg/dL (normal range: 3.4-8.8 mg/dL), C-reactive protein was 8.0 mg/dL (normal range: < 0.8 mg/dL), and erythrocyte sedimentation rate was 73 mm/hr (normal range: < = 12 mm/hr), which were all significantly elevated. The rheumatoid factor was minimally elevated at 15 IU/mL (normal range: < 14 IU/mL). Cyclic citrullinated peptide and anti-nuclear antibody levels were within the normal range (Table 1). 

**Table 1:** Laboratory values on initial presentation

Laboratory testing	Results	Reference range	Value interpretation
Serum urate	10.2 mg/dL	3.4-8.8 mg/dL	Elevated
C-reactive protein	8.0 mg/dL	< 0.8 mg/dL	Elevated
Erythrocyte sedimentation rate	73 mm/hr	< =12 mm/Hr	Normal
Rheumatoid factor	15 IU/mL	< 14 IU/mL	Minimally elevated
Cyclic citrullinated peptide	3 units	< 19 units	Normal
Anti-nuclear antibody	< 1:40 titer	< 1:40 titer	Normal

Methylprednisolone dosepak was prescribed, and despite completion, his symptoms persisted with minimal improvement. Colchicine was then initiated at 1.2 mg once, followed by 0.6 mg once one hour later. Colchicine was then continued at 0.6 mg daily for maintenance. 

**Figure 1:**
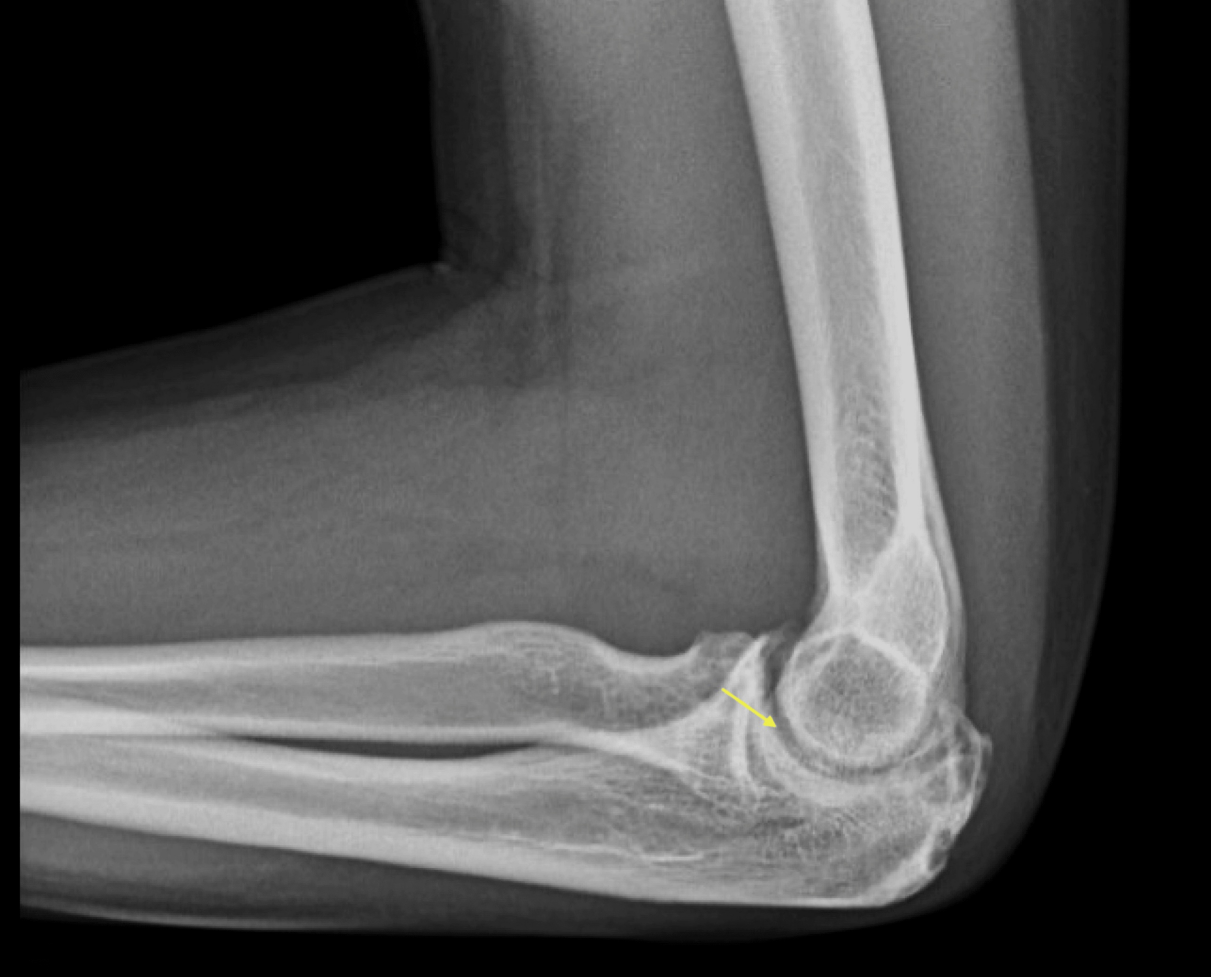
Left elbow osteoarthritis with erosion (arrow)

**Figure 2:**
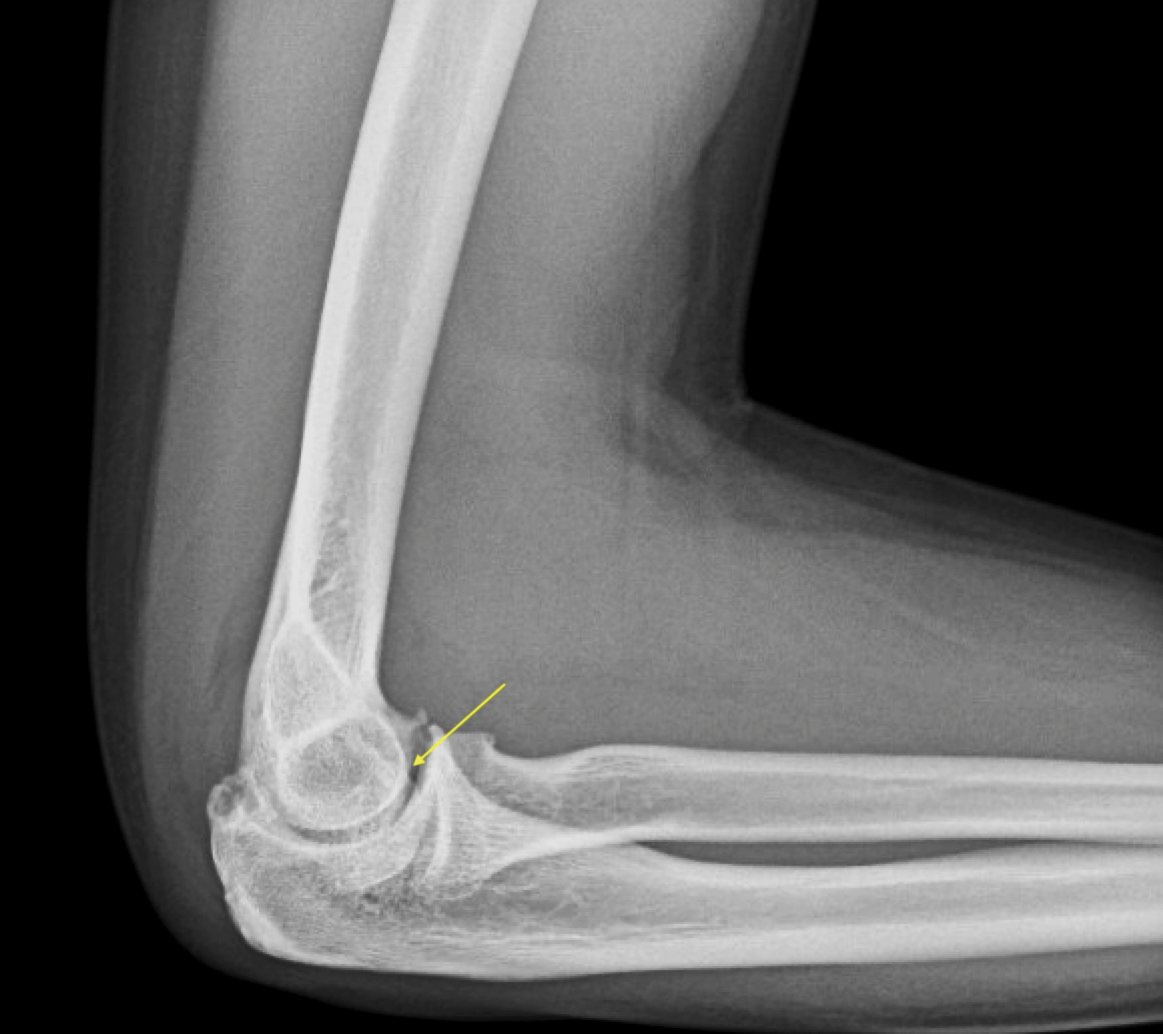
Right elbow osteoarthritis with erosion and effusion (arrow)

After two weeks on colchicine, he presented to the rheumatologist with evidence of tenderness in multiple joints, including the hands and elbows. Edema was noted in the right third metacarpophalangeal, fifth proximal interphalangeal joint, and right elbow. The X-ray of the elbows revealed moderate bilateral arthritis with associated erosions (Figures 1, 2).

**Figure 3:**
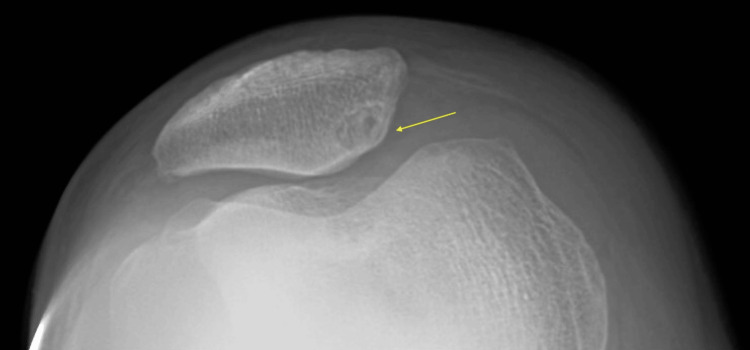
Right knee with osteoarthritis and a subarticular degenerative cyst of the medial facet (arrow)

In addition, there was a right elbow effusion (Figure 2). These findings suggested possible gout. The X-ray of his hands revealed mild bilateral osteoarthritis of multiple joints, including the first carpometacarpal joint and distal interphalangeal joints. Otherwise, the findings were normal. The X-ray of his right knee confirmed a large joint effusion in addition to a large subarticular degenerative cyst about the medial facet and early osteoarthritis (Figure 3).

The patient was started on a course of prednisone: 40 mg daily for seven days. The colchicine dose remained at 0.6 mg once daily while nonsteroidal anti-inflammatory drugs (NSAIDs) were avoided due to the development of chronic kidney disease. After confirming the patient tested negative for HLA-B58.01, he was started on allopurinol 100 mg daily to achieve the goal of uric acid under 6.0 mg/dL. 

## Discussion

Gout may present as acute or chronic inflammatory arthritis. Tophaceous gout involves a severe form of gout with the formation of uric acid crystal deposits, known as tophi, under the skin or within joints. This can lead to significant pain, joint deformity, and damage. Hyperuricemia is defined as a serum urate level of over 7.0 mg/dL in men and over 5.7 mg/dL in women [1]. Associated comorbidities of gout include cardiovascular disease, chronic kidney disease, gout nephropathy, metabolic syndrome, and type 2 diabetes. Early-onset hyperuricemia is considered to be more likely attributed to genetic variants involved in impairments in urate export compared to dietary habits. Familial gout is reported more frequently in those who present with an early age of onset [3]. Known provoking factors that may induce a gout flare and involve an increase in serum urate include high ambient temperatures, humidity, dehydration, starvation involving ketoacidosis, and consumption of high-fructose corn syrup and alcohol [4]. Other risk factors include older age, male sex, obesity, and chronic kidney disease. Medications that may increase serum urate include, but are not limited to, loop and thiazide diuretics, low-dose aspirin, and calcineurin inhibitors [1]. Purine-rich foods, including red meat and seafood, have been shown to increase the risk of recurrent gout attacks by almost five times among patients with gout. Consumption of higher levels of purine, specifically from animal sources, is associated with elevated serum urate levels. The purine content in processed, dried, and canned fish is higher compared to that of fresh fish [4,5]. 

The clinical presentation may be sufficient to suspect gout, as it is known to be commonly associated with an acutely inflamed joint, particularly the first metatarsophalangeal joint. While this can occur in the setting of hyperuricemia, the serum urate level may decrease during a gout flare. The differential diagnosis should include septic arthritis, psoriatic arthritis, rheumatoid arthritis, and calcium pyrophosphate crystal deposition disease. Synovial fluid obtained from joint aspiration can confirm monosodium urate crystals. Under polarized light with light microscopy, needle- or rod-shaped crystals are seen with negative birefringence with yellow or blue color. This differs from calcium pyrophosphate crystals, which are weakly positively birefringent with polymorphic forms under polarized light. If imaging is considered, plain radiographs can be done, which may identify bony erosions. However, it is likely to appear normal in acute episodes [1,6]. An alternative diagnostic test involves dual-energy CT imaging, with its high sensitivity and less invasive method of detecting monosodium urate crystals [7]. 

For an acute gout attack, the goal of treatment would be to reduce serum urate levels to under 6.0 mg/dL and target the inflammatory reaction involving an innate immune response and activation of cytokines against gout-containing monosodium urate crystals. This can be achieved with anti-inflammatory treatment, such as oral NSAIDs, colchicine, or glucocorticoids, which should be started within 12 to 24 hours of the onset of the acute gout attack. Colchicine may be an effective option, given its role in preventing the activation and migration of neutrophils involved in the transport of monosodium urate crystals. It may also be used for prophylaxis for the prevention of recurrent flares. Side effects include nausea, vomiting, or diarrhea. Importantly, NSAIDs and colchicine are contraindicated in severe chronic kidney disease with an estimated glomerular filtration rate of less than 30 mL/min [8]. 

According to the American College of Rheumatology, urate-lowering therapy is strongly recommended for those with at least two flares annually, at least one subcutaneous tophus, or evidence of joint damage secondary to gout on imaging. Earlier initiation of treatment is also advised. It is not recommended after a first incident of gout, which was not complicated. However, if the patient has moderate to severe chronic kidney disease, serum urate concentration over 9 mg/dL, or urolithiasis, urate-lowering therapy is recommended, given the increased risk of gout progression and development of tophi [9]. Alternatively, it may be more practical to guide the treatment decision with the intent to lower urate levels to under 6.0 mg/dL while incorporating the preventative lifestyle changes. This can prevent the development of bony erosion and tophaceous deposits, which can lead to joint impairment and deformity. Allopurinol, a xanthine oxidase inhibitor that prevents uric acid synthesis, is a common choice for long-term use. It is usually started at 100 mg daily in patients with a creatinine clearance of over 60 mL/minute. The dose will need to be adjusted in patients with chronic kidney disease. After initiating treatment, serum urate levels can be monitored every two to three weeks, after which the allopurinol dose can be adjusted to reach the goal serum urate levels. Prior to initiating treatment, patients who are of Asian or African descent are evaluated for the HLA-B*5801 allele. If positive, they may be at risk for severe cutaneous adverse reactions. If a xanthine oxidase inhibitor is needed, febuxostat can be considered. The dosing for all these treatments may need to be adjusted in the presence of chronic kidney disease [1,6,10].

## Conclusions

Polyarticular gout can be easily investigated with a uric acid level in the setting of a high index of suspicion. In this case, the patient was considered to have early-onset polyarticular gout, given that the time of onset was before the age of 40. The prevalence of hyperuricemia in early-onset gout is lower than the prevalence of gout in the general population with hyperuricemia. However, in countries with a higher prevalence of gout, there is a higher prevalence of hyperuricemia in the younger population. This suggests the possibility of genetic predispositions associated with innate immunity, which can increase the reactivity to monosodium urate crystals. This continues to be an area of study. Dietary interventions, then, may not be expected to have a significant impact on reducing serum urate levels. However, reducing purine-rich food and beverages may prevent the associated medical comorbidities while reducing the risk of cardiovascular disease. Preventative strategies should then be prioritized. This may also include weight loss, exercise, avoiding high fructose corn syrup, and limiting alcohol intake. The treatment approach for an acute attack and for urate-lowering therapy remains the same in all patients with gout, regardless of age.
